# Congenital Pelger-Huët anomaly in a Danish/Swedish Farmdog: Case Report

**DOI:** 10.1186/1751-0147-53-14

**Published:** 2011-03-01

**Authors:** Janina Lukaszewska, Robin W Allison, Julita Stepkowska

**Affiliations:** 1 Veterinary Health Center, Wroclaw, Poland; 2 Department of Veterinary Pathobiology, Oklahoma State University, Center for Veterinary Health Sciences, Stillwater, Oklahoma, USA; 3 Veterinary Clinic for Small Animals "Lancet", Warsaw, Poland

## Abstract

A 13 year old Danish/Swedish Farmdog from Denmark was evaluated in a veterinary clinic in Warsaw, Poland for evaluation of an orthopedic problem. Radiographs revealed spondylosis and degenerative vertebral disease, which responded to treatment with anti-inflammatory medications. A predominance of hyposegmented neutrophils and eosinophils containing condensed chromatin and normal cytoplasm were identified on a routine CBC. Follow-up blood film evaluations over the course of 12 months confirmed that the hyposegmented granulocytes persisted. The majority of neutrophils contained Grade 2 nuclei (slightly indented), and the mean nuclear score varied from 1.9 to 2.3. Pelger-Huët anomaly (PHA), presumably congenital, was diagnosed based on persistent hyposegmented granulocytes in the absence of an underlying cause for acquired PHA; genetically related dogs were unavailable for testing to confirm vertical transmission. To the authors' knowledge this is the first report of PHA in a Danish/Swedish Farmdog.

## Background

Morphologic evaluation of leukocytes by microscopic examination of a blood film is an important component of the complete blood count (CBC). Even when total leukocyte numbers are within reference intervals, identification of immature hyposegmented neutrophils in increased numbers (a left shift) signifies an inflammatory leukogram. Hyposegmentation of neutrophils also occurs with Pelger-Huët anomaly (PHA). Congenital Pelger-Huët anomaly is a familial defect in granulocyte nuclear segmentation first described in humans in The Netherlands by Dutch physicians, K. Pelger and G. Huët in 1928 and 1932, respectively.[1,2] Mutations in the lamin B receptor (LBR) have recently been identified as the cause of PHA in humans.[3] LBR is a conserved integral membrane protein of the nuclear envelope that interacts with lamin B and heterochromatin, and has been shown to be required for the normal morphologic maturation of granulocytes.[4,5] Granulocyte function in affected individuals appears to be normal.[4,6-8] The hereditary form of PHA must be differentiated from pseudo-PHA, a temporary condition acquired secondary to an underlying disease or drug administration.[9] The mechanism underlying granulocyte hypolobulation in pseudo-PHA remains to be elucidated, but reduced expression of LBR has been postulated.[10] Here we report apparently congenital PHA in an aged Danish/Swedish Farmdog.

## Case Presentation

**Figure 1:**
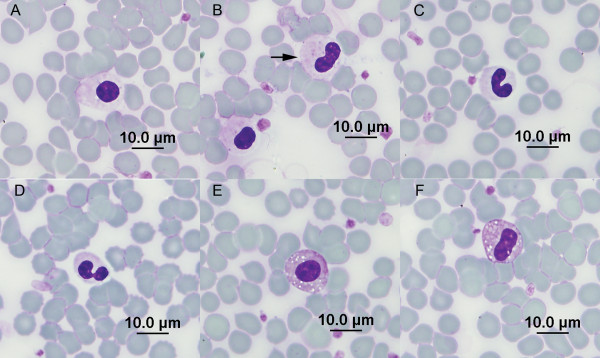
**Peripheral blood neutrophils and eosinophils from a Danish dog with Pelger-Huët anomaly**. (A) Grade 1 nucleus, neutrophil. (B) Grade 1 nucleus and grade 2 nucleus (arrow), neutrophils. (C) Grade 3 nucleus, neutrophil. (D) Grade 4 nucleus, neutrophil. (E) Grade 1 nucleus, eosinophil. (F) Grade 2 nucleus, eosinophil. May-Grunwald-Giemsa stain.

A 13 year old intact male Danish/Swedish Farmdog, born in Denmark, was brought to a private clinic in Warsaw, Poland for evaluation of an orthopedic problem (difficulty negotiating stairs). Physical examination was unremarkable with the exception of pain elicited on compression of the sacrolumbar spine. The dog had a 2-year history of cardiac insufficiency, and was being treated with pimobendan (0.25 mg/kg BID), benazepril (0.25 mg/kg SID), spironolactone (2 mg/kg BID) and furosemide (1 mg/kg PRN). Radiographs were obtained of the spine, and a CBC (ABC Vet, Horiba ABX, Montpellier, France), biochemistry profile (Reflovet Plus, Scil Animal Care Company, Viernheim, Germany) and urinalysis were obtained. Radiographs revealed moderate spondylosis of the thoracolumbar and lumbosacral spine, with mild degenerative changes of the lumbar vertebrae. Biochemical abnormalities were limited to increased serum activity of alanine aminotransferase (ALT)(197 U/L; reference interval <47 U/L), aspartate aminotransferase (AST) (38 U/L; reference interval <19 U/L), and alkaline phosphatase (ALP) (642 U/L; reference interval <68 U/L). Liver size was normal and no parenchymal lesions were identified on ultrasound examination, thus hepatic enzyme abnormalities were interpreted as secondary to cardiac insufficiency. The urinalysis was normal. Results of the CBC were normal except for exclusively hyposegmented neutrophils and eosinophils identified on the blood film (Figure 1) and a mild monocytosis. The dog was treated with tolfenamic acid (4 mg/kg SID for 5 days) for spinal pain, and clinical signs improved within a few days.

To further evaluate the hyposegmented granulocytes, CBCs were repeated after two, six and twelve months. Results were always within normal limits, except that no granulocytes containing normal segmented nuclei were observed on any blood film (Table 1 and 2). One hundred consecutive neutrophil nuclei were graded based on a classification system published by Bowles, et al.[11] (Table 2). Grade 1 nuclei are round or oval, Grade 2 are slightly indented, Grade 3 are band-shaped, Grade 4 have two lobes, Grade 5 have three lobes, etc. Mean nuclear scores (MNS) were determined by multiplying the grade by the number of cells in each category, and dividing the sum by the number of observations (100). The lower the MNS, the less segmentation is present. At each time point Grade 2 nuclei predominated, followed by Grade 1 nuclei in 3 of 4 evaluations. The greatest number of nuclear lobes identified was two. Eosinophils were almost exclusively Grade 1 and 2; there were rare Grade 3 nuclei. Based on the characteristic nuclear changes in neutrophils and eosinophils that persisted over 12 months and the absence of an underlying condition, a diagnosis of congenital Pelger-Huët anomaly was made.

**Table 1:** Results of serial CBCs from a Danish/Swedish Farmdog with Pelger-Huët anomaly.

Analyte	Day 1	2 months	6 months	12 months	Reference interval
RBC (10^12^/L)	5.38	6.02	5.90	4.96	5.4 - 8.9
HGB (g/L)	131	141	137	128	130 -190
HCT (L/L)	0.37	0.39	0.40	0.34	0.37 - 0.55
MCV (fL)	68	65	73	68	60 -74
MCH (pg)	24.3	23.4	23.7	25.8	22 -27
MCHC (g/L)	359	361	325	378	340 -360
Platelets (10^9^/L)	260	421	209	401	160 -500
WBC (10^9^/L)	13.63	8.70	9.76	6.79	7.0 -17.0
Neutrophils (10^9^/L)	9.96	6.88	6.56	4.35	3.0-11.8
Lymphocytes (10^9^/L)	1.09	0.61	1.74	1.15	1.0-4.8
Eosinophils (10^9^/L)	0.95	0.34	0.58	0.61	0.2-1.5
Monocytes (10^9^/L)	1.63	0.87	0.88	0.68	1.0-1.3

**Table 2:** Neutrophil nuclear segmentation grades* and mean nuclear scores (MNS) in serial CBCs from a Danish/Swedish Farmdog with Pelger-Huët anomaly.

	Day 1	2 months	6 months	12 months
Grade 1 (%)	30	34	14	21
Grade 2 (%)	53	44	53	36
Grade 3 (%)	16	20	29	37
Grade 4 (%)	1	2	4	6
MNS	1.9	1.9	2.2	2.3

* 100 consecutive neutrophil nuclei were counted and categorized to determine nuclear segmentation grade; see Figure 1 for examples.

Since first discovered, congenital PHA has been reported in a variety of animal species including dogs, cats, rabbits, mice, and horses.[11-15] The classic features recognized on blood film evaluation are hypolobulated granulocytes (neutrophils, eosinophils, and basophils) containing mature condensed nuclear chromatin. Nuclei are typically round, oval, rod or band shaped, or bilobed. Hypolobulation of monocytes and megakaryocytes has also been reported.[16] The acquired pseudo-PHA occurs in people but reports in domestic animals are limited to cattle; one report in a dog was later found to be congenital.[16-19] Persistence of hyposegmented granulocytes over time in the absence of an underlying pathologic state may be considered de facto evidence for the congenital form of PHA, however identification of related individuals with the same morphologic abnormalities is required for definitive diagnosis.

In the current case, marked hypolobulation of neutrophils and eosinophils was documented over the course of a year in the absence of underlying disease and while the patient was not receiving medication. Unfortunately, this dog has no offspring and efforts to locate siblings in Denmark were unsuccessful; thus the diagnosis of congenital PHA is presumed rather than definitive. The Danish/Swedish Farmdog, recognized as a breed in Denmark and Sweden in 1987, is a small dog resembling a terrier but related to the pinscher family. Although PHA has been documented in many different dog breeds, there has been only one previous report from Europe.[20,21]

## Conclusions

To the authors' knowledge, the current case is the first in a Danish/Swedish Farmdog and the second report of canine PHA to originate from Europe. Recognizing the features of PHA on blood film evaluation, particularly in an aged dog with possible underlying disease, is important to avoid misidentification of an inflammatory left shift.

## Competing interests

The authors declare that they have no competing interests.

## Authors' contributions

JL and JS treated the patient, obtained blood samples, and evaluated blood films. JL graded granulocyte nuclei. RWA evaluated blood films, obtained photomicrographs, and calculated mean nuclear scores. JL and RWA drafted the manuscript. All authors read and approved the final manuscript.

## References

[B1] Huet GJ Ueber eine bisher unbekannte familiaere Anomalie der Leukocyten [About a previously unknown familial anomaly of leukocytes] Klin Wochenschr 1932 11 1264 1266 (In German) 10.1007/BF01758934

[B2] Pelger K Demonstratie van een paar zeldzaam voorkomende typen van bloedlichaampjes en bespreking der patienten [Demonstration of rare types of blood cells and discussion of patients] Ned Tijdschr Geneeskd 1928 72 1178 (In Deutsch)

[B3] Hoffmann K Dreger CK Olins AL Olins DE Shultz LD Lucke B Karl H Kaps R Muller D Vaya A Mutations in the gene encoding the lamin B receptor produce an altered nuclear morphology in granulocytes (Pelger- Huët anomaly) Nat Genet 2002 31 410 414 12118250 10.1038/ng925

[B4] Cohen TV Klarmann KD Sakchaisri K Cooper JP Kuhns D Anver M Johnson PF Williams SC Keller JR Stewart CL The lamin B receptor under transcriptional control of C/EBPepsilon is required for morphological but not functional maturation of neutrophils Hum Mol Genet 2008 17 2921 2933 10.1093/hmg/ddn191 18621876 PMC2536505

[B5] Hoffmann K Sperling K Olins AL Olins DE The granulocyte nucleus and lamin B receptor: avoiding the ovoid Chromosoma 2007 116 227 235 10.1007/s00412-007-0094-8 17245605

[B6] Speeckaert MM Verhelst C Koch A Speeckaert R Lacquet F Pelger- Huët anomaly: a critical review of the literature Acta Haematol 2009 121 202 206 10.1159/000220333 19468205

[B7] Latimer KS Kircher IM Lindl PA Dawe DL Brown J Leukocyte function in Pelger- Huët anomaly of dogs Journal of leukocyte biology 1989 45 301 310 2649629 10.1002/jlb.45.4.301

[B8] Latimer KS Prasse KW Neutrophilic movement of a Basenji with Pelger- Huët anomaly American journal of veterinary research 1982 43 525 527 7073070

[B9] Dusse LM Moreira AM Vieira LM Rios DR Morais ESRM Carvalho MD Acquired Pelger- Huët: What does it really mean? Clin Chim Acta 2010 10.1016/j.cca.2010.07.011 20691170

[B10] Best S Salvati F Kallo J Garner C Height S Thein SL Rees DC Lamin B-receptor mutations in Pelger- Huët anomaly Br J Haematol 2003 123 542 544 10.1046/j.1365-2141.2003.04621.x 14617022

[B11] Bowles CA Alsaker RD Wolfle TL Studies of the Pelger- Huët anomaly in foxhounds The American journal of pathology 1979 96 237 248 464021 PMC2042355

[B12] Latimer KS Rakich PM Thompson DF Pelger- Huët anomaly in cats Veterinary pathology 1985 22 370 374 4035941 10.1177/030098588502200412

[B13] Gill AF Gaunt S Sirninger J Congenital Pelger- Huët anomaly in a horse Veterinary clinical pathology/American Society for Veterinary Clinical Pathology 2006 35 460 462 17123255 10.1111/j.1939-165x.2006.tb00165.x

[B14] Green MC Shultz LD Nedzi LA Abnormal nuclear morphology of leukocytes in the mouse mutant ichthyosis Transplantation 1975 20 172 175 10.1097/00007890-197508000-00014 1101484

[B15] Nachtsheim H The Pelger-anomaly in man and rabbit; a mendelian character of the nuclei of the leucocytes J Hered 1950 41 131 137 15436969 10.1093/oxfordjournals.jhered.a106108

[B16] Latimer KS Duncan JR Kircher IM Nuclear segmentation, ultrastructure, and cytochemistry of blood cells from dogs with Pelger- Huët anomaly Journal of comparative pathology 1987 97 61 72 10.1016/0021-9975(87)90128-9 2435771

[B17] Carper HA Pseudo-Pelger neutrophils in the cow Vet Med Small Anim Clin 1965 60 997 998 5173814

[B18] Osburn BI Glenn BL Acquired Pelger Huët anomaly in cattle Journal of the American Veterinary Medical Association 1968 152 11 16 5688703

[B19] Shull RM Powell D Acquired hyposegmentation of granulocytes (pseudo-Pelger- Huët anomaly) in a dog Cornell Vet 1979 69 241 247 477323

[B20] Latimer KS Campagnoli RP Danilenko DM Pelger- Huët anomaly in Australian Shepherds: 87 cases (1991-1997) Comparative Haematology International 2000 10 9 13 10.1007/s005800070021

[B21] Kiss M Komar G Jr Pelger-Huët'sche Kernanomalie der Leukozyten bei einem Hunde [Pelger-Huet nuclear anomaly in leukocytes in a dog] Berl Munch Tierarztl Wochenschr 1967 80 474 476 In German with summary in English 5628116

